# Poly[[triaqua­tri-μ_5_-tartrato-dilanthanum(III)] dihydrate]

**DOI:** 10.1107/S160053680801756X

**Published:** 2008-06-19

**Authors:** Liu Shi-Zhu

**Affiliations:** a School of Chemistry and Environment, South China Normal University, Guangzhou 510006, People’s Republic of China

## Abstract

In the title polymer, {[La_2_(C_4_H_4_O_6_)_3_(H_2_O)_3_]·2H_2_O}_*n*_, two symmetry-independent La^III^ ions are nine-coordinated and display a distorted monocapped square-anti­prismatic geometry. One is coordinated by seven O atoms from four tartrate ligands and two water mol­ecules, the other by eight O atoms from five tartrate ligands and one water mol­ecule. The three tartrate ligands in the asymmetric unit act identically as μ_5_-ligands, which link lanthanum centres to form a three-dimensional coordination framework. An extensive network of hydrogen bonds is observed in the crystal structure, involving two uncoordinated water mol­ecules, one of which is disordered over two positions, with occupancies of 0.550 (13) and 0.450 (13).

## Related literature

For related literature, see: Yaghi *et al.* (1998 ▶, 2003 ▶); Serre *et al.* (2004 ▶); James *et al.* (2003 ▶).
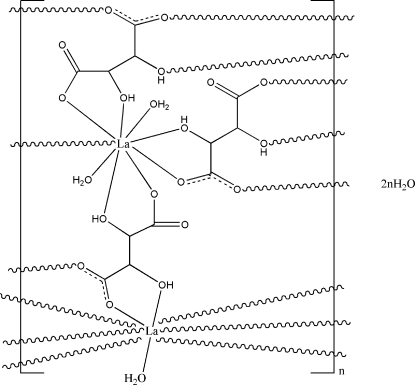


## Experimental

### 

#### Crystal data

[La_2_(C_4_H_4_O_6_)_3_(H_2_O)_3_]·2H_2_O
*M* *_r_* = 812.12
Monoclinic, 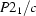

*a* = 12.6271 (2) Å
*b* = 12.9273 (2) Å
*c* = 16.6556 (3) Å
β = 127.801 (1)°
*V* = 2148.22 (6) Å^3^
*Z* = 4
Mo *K*α radiation
μ = 4.04 mm^−1^
*T* = 296 (2) K
0.25 × 0.21 × 0.18 mm

#### Data collection

Bruker APEXII area-detector diffractometer
Absorption correction: multi-scan (*SADABS*; Sheldrick, 1996 ▶) *T*_min_ = 0.372, *T*_max_ = 0.480
19115 measured reflections
3771 independent reflections
3226 reflections with *I* > 2σ(*I*)
*R*_int_ = 0.047

#### Refinement

*R*[*F* ^2^ > 2σ(*F* ^2^)] = 0.026
*wR*(*F* ^2^) = 0.057
*S* = 1.05
3771 reflections
344 parameters
H-atom parameters constrained
Δρ_max_ = 0.87 e Å^−3^
Δρ_min_ = −0.65 e Å^−3^

### 

Data collection: *SMART* (Bruker, 2004 ▶); cell refinement: *SAINT* (Bruker, 2004 ▶); data reduction: *SAINT*; program(s) used to solve structure: *SHELXS97* (Sheldrick, 2008 ▶); program(s) used to refine structure: *SHELXL97* (Sheldrick, 2008 ▶); molecular graphics: *SHELXTL* (Sheldrick, 2008 ▶); software used to prepare material for publication: *SHELXTL*.

## Supplementary Material

Crystal structure: contains datablocks I, global. DOI: 10.1107/S160053680801756X/bh2174sup1.cif

Structure factors: contains datablocks I. DOI: 10.1107/S160053680801756X/bh2174Isup2.hkl

Additional supplementary materials:  crystallographic information; 3D view; checkCIF report

## Figures and Tables

**Table 1:** Hydrogen-bond geometry (Å, °)

*D*—H⋯*A*	*D*—H	H⋯*A*	*D*⋯*A*	*D*—H⋯*A*
O3—H3*A*⋯O8^i^	0.82	1.87	2.677 (4)	169
O4—H4⋯O18^ii^	0.82	1.80	2.619 (4)	174
O9—H9⋯O16	0.82	2.46	3.177 (5)	147
O10—H10*A*⋯O8^iii^	0.82	1.94	2.745 (4)	167
O15—H15⋯O2^i^	0.82	1.82	2.632 (4)	175
O16—H16⋯O4*WA*	0.82	1.89	2.667 (9)	158
O16—H16⋯O4*WB*	0.82	1.94	2.708 (9)	157

Symmetry codes: (i) 
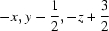
; (ii) 
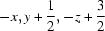
; (iii) 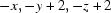.

**Fig. 1.**
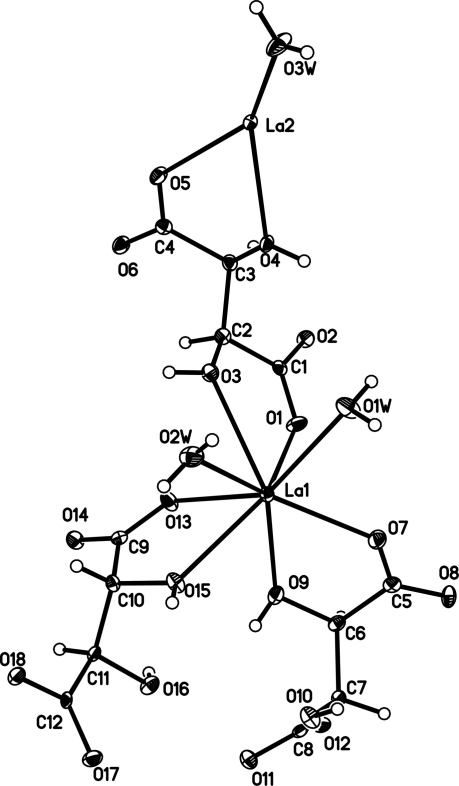
The structure of (I), showing the atomic numbering scheme. Non-H atoms are shown with 30% probability displacement ellipsoids.

**Fig. 2.**
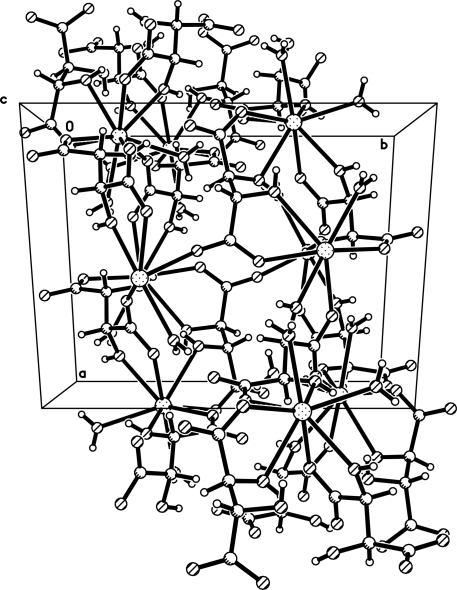
A packing view of (I), showing the inter and intramolecular hydrogen bonds.

## supplementary crystallographic information

### Comment

The use of multifunctional organic linker molecules to polymerize metal centers into open-framework materials has led to the development of a rich field of chemistry (Yaghi *et al.*, 1998, 2003; Serre *et al.*, 2004; James, 2003) owing to the potential applications of these materials in catalysis, separation, gas storage and molecular recognition. Among such novel open-framework materials, lanthanide oxalates are particularly noteworthy. The wide variety of coordination modes of the tartarate anion permits the use of metal-tartarate units as excellent building blocks to construct a great diversity of frameworks ranging from discrete oligomeric entities to one-, two- and three-dimensional networks. Recently, we obtained the title La^III^ polymer, (I), and its crystal structure is reported here.

In the asymmetric unit of (I), two symmetry independent La^III^ ions are nine-coordinated and display a distorted monocapped square antiprism geometry. One is coordinated by seven O atoms from four tartarate ligands and two coordinated water molecules, the other is defined by eight O atoms from five tartarate ligands and one coordinated water molecule (Fig. 1). All three unique tartarate ligands only act as one type of coordination mode, which link lanthanum centres to form a three-dimensional coordination framework (Fig. 2). The shortest La···La separations in the solid are 6.207 (2), 6.520 (3) and 6.535 (2) Å. The voids between the individual metal complex units are filled with classical hydrogen bonded (Table 1) interstitial disordered water molecules.

### Experimental

A mixture of La_2_O_3_ (0.5 mmol), tartaric acid (1.5 mmol) and H_2_O (10 ml) in the presence of HClO_4_ (0.385 mmol) was stirred vigorously for 20 min and then sealed in a Teflon-lined stainless-steel autoclave (20 ml, capacity). The autoclave was heated to 433 K and maintained at this temperature for 7 days, and then cooled to room temperature at 5 K.h^-1^. The crystals were obtained in *ca.* 46% yield based on La.

### Refinement

Lattice water molecule O4W is disordered over two sites, O4WA and O4WB, with refined occupancies of 0.450 (13) and 0.550 (13), respectively. Water H atoms were located in a difference map and refined isotropically with *U*_iso_(H) = 1.5*U*_eq_(O) and a regularized geometry: O—H bond lengths were restrained to 0.82 (2)/0.85 (1) Å and H···H separations were restrained to 1.35 (2) Å for coordinated and 1.39 Å for interstitial water molecules. In the final cycles, a riding model was applied for all water H atoms. All other H atoms were placed in calculated positions with a C—H distance of 0.98 Å and O—H distance of 0.82 Å, and refined using a riding model with *U*_iso_(H) = 1.2*U*_eq_(C) for CH groups and *U*_iso_(H) = 1.5*U*_eq_(O) for hydroxyl groups.

### Crystal data

**Table:**

[La_2_(C_4_H_4_O_6_)_3_(H_2_O)_3_]·2H_2_O	*F*_000_ = 1568
*M**_r_* = 812.12	*D*_x_ = 2.511 Mg m^−^^3^
Monoclinic, *P*2_1_/*c*	Mo *K*α radiation λ = 0.71073 Å
Hall symbol: -P 2ybc	Cell parameters from 15678 reflections
*a* = 12.6271 (2) Å	θ = 1.4–28.0º
*b* = 12.9273 (2) Å	µ = 4.04 mm^−^^1^
*c* = 16.6556 (3) Å	*T* = 296 (2) K
β = 127.801 (1)º	Block, colourless
*V* = 2148.22 (6) Å^3^	0.25 × 0.21 × 0.18 mm
*Z* = 4	

### Data collection

**Table:**

Bruker APEXII area-detector diffractometer	3771 independent reflections
Radiation source: fine-focus sealed tube	3226 reflections with *I* > 2σ(*I*)
Monochromator: graphite	*R*_int_ = 0.047
*T* = 296(2) K	θ_max_ = 25.2º
φ and ω scans	θ_min_ = 2.0º
Absorption correction: multi-scan(SADABS; Sheldrick, 1996)	*h* = −15→15
*T*_min_ = 0.372, *T*_max_ = 0.480	*k* = −15→14
19115 measured reflections	*l* = −19→19

### Refinement

**Table:**

Refinement on *F*^2^	Secondary atom site location: difference Fourier map
Least-squares matrix: full	Hydrogen site location: inferred from neighbouring sites
*R*[*F*^2^ > 2σ(*F*^2^)] = 0.026	H-atom parameters constrained
*wR*(*F*^2^) = 0.057	*w* = 1/[σ^2^(*F*_o_^2^) + (0.0203*P*)^2^ + 3.8165*P*] where *P* = (*F*_o_^2^ + 2*F*_c_^2^)/3
*S* = 1.05	(Δ/σ)_max_ = 0.001
3771 reflections	Δρ_max_ = 0.87 e Å^−^^3^
344 parameters	Δρ_min_ = −0.65 e Å^−^^3^
Primary atom site location: structure-invariant direct methods	Extinction correction: none

### Fractional atomic coordinates and isotropic or equivalent isotropic displacement parameters (Å^2^)

**Table:**

	*x*	*y*	*z*	*U*_iso_*/*U*_eq_	Occ. (<1)
C1	−0.0900 (4)	0.9566 (4)	0.5698 (3)	0.0155 (10)	
C2	−0.1083 (4)	0.8523 (4)	0.5199 (3)	0.0177 (10)	
H2	−0.0295	0.8416	0.5224	0.021*	
C3	−0.2308 (4)	0.8455 (4)	0.4086 (3)	0.0176 (10)	
H3	−0.2432	0.9136	0.3782	0.021*	
C4	−0.2140 (4)	0.7672 (4)	0.3482 (3)	0.0174 (10)	
C5	0.0357 (5)	1.0235 (4)	0.9197 (3)	0.0183 (10)	
C6	0.1840 (4)	0.9984 (4)	0.9767 (3)	0.0184 (10)	
H6	0.2249	1.0587	0.9697	0.022*	
C7	0.2635 (4)	0.9782 (3)	1.0918 (3)	0.0165 (10)	
H7	0.2333	1.0265	1.1193	0.020*	
C8	0.4144 (4)	0.9950 (4)	1.1463 (3)	0.0172 (10)	
C9	0.2706 (4)	0.7265 (4)	0.8239 (3)	0.0171 (10)	
C10	0.2636 (4)	0.6536 (4)	0.8918 (3)	0.0152 (10)	
H10	0.2424	0.5841	0.8623	0.018*	
C11	0.3977 (4)	0.6497 (3)	0.9974 (3)	0.0150 (10)	
H11	0.4687	0.6446	0.9899	0.018*	
C12	0.4096 (4)	0.5570 (3)	1.0589 (3)	0.0137 (10)	
La1	−0.02226 (2)	0.81431 (2)	0.77118 (2)	0.01422 (8)	
La2	−0.54797 (2)	0.73846 (2)	0.22545 (2)	0.01381 (8)	
O1	−0.0471 (3)	0.9566 (3)	0.6612 (2)	0.0248 (8)	
O2	−0.1144 (3)	1.0376 (2)	0.5208 (2)	0.0207 (7)	
O3	−0.1053 (3)	0.7743 (2)	0.5816 (2)	0.0221 (7)	
H3A	−0.0661	0.7267	0.5776	0.033*	
O4	−0.3519 (3)	0.8206 (2)	0.3936 (2)	0.0184 (7)	
H4	−0.3650	0.8645	0.4222	0.028*	
O5	−0.3178 (3)	0.7172 (2)	0.2791 (2)	0.0221 (8)	
O6	−0.1040 (3)	0.7609 (3)	0.3649 (2)	0.0232 (8)	
O7	−0.0495 (3)	0.9637 (3)	0.8484 (2)	0.0255 (8)	
O8	0.0074 (3)	1.1045 (2)	0.9438 (3)	0.0226 (8)	
O9	0.1947 (3)	0.9140 (3)	0.9266 (3)	0.0294 (8)	
H9	0.2372	0.8703	0.9710	0.044*	
O10	0.2457 (3)	0.8752 (2)	1.1112 (2)	0.0237 (8)	
H10A	0.1714	0.8711	1.0973	0.036*	
O11	0.4892 (3)	0.9167 (2)	1.1844 (2)	0.0214 (7)	
O12	0.4517 (3)	1.0853 (2)	1.1460 (2)	0.0213 (7)	
O13	0.1759 (3)	0.7910 (3)	0.7713 (3)	0.0256 (8)	
O14	0.3687 (3)	0.7181 (3)	0.8235 (2)	0.0235 (8)	
O15	0.1587 (3)	0.6864 (2)	0.8953 (2)	0.0197 (7)	
H15	0.1438	0.6427	0.9228	0.030*	
O16	0.4191 (3)	0.7419 (2)	1.0529 (2)	0.0212 (7)	
H16	0.4526	0.7832	1.0370	0.032*	
O17	0.4440 (3)	0.5724 (2)	1.1455 (2)	0.0238 (8)	
O18	0.3836 (3)	0.4696 (2)	1.0164 (2)	0.0194 (7)	
O1W	−0.2694 (3)	0.8479 (3)	0.6519 (3)	0.0322 (9)	
H1W	−0.3029	0.8425	0.6809	0.048*	
H2W	−0.3074	0.8967	0.6129	0.048*	
O2W	−0.1015 (3)	0.6276 (3)	0.7134 (2)	0.0307 (8)	
H4W	−0.1775	0.6138	0.6932	0.046*	
H3W	−0.0543	0.5815	0.7543	0.046*	
O3W	−0.7660 (3)	0.6479 (3)	0.1489 (3)	0.0363 (10)	
H6W	−0.8076	0.6421	0.1715	0.054*	
H5W	−0.8025	0.6085	0.1004	0.054*	
O5W	0.6940 (6)	0.5141 (5)	0.6746 (7)	0.139 (3)	
H10W	0.6504	0.5618	0.6312	0.208*	
H9W	0.6446	0.4937	0.6900	0.208*	
O4WA	0.4665 (13)	0.9039 (8)	0.9799 (8)	0.056 (5)	0.450 (13)
H4WA	0.4304	0.8494	0.9818	0.084*	0.450 (13)
H4WB	0.4877	0.8889	0.9417	0.084*	0.450 (13)
O4WB	0.5902 (11)	0.8436 (8)	1.0338 (7)	0.072 (4)	0.550 (13)
H4WC	0.5854	0.8295	0.9818	0.108*	0.550 (13)
H4WD	0.5400	0.8961	1.0169	0.108*	0.550 (13)

### Atomic displacement parameters (Å^2^)

**Table:**

	*U*^11^	*U*^22^	*U*^33^	*U*^12^	*U*^13^	*U*^23^
C1	0.008 (2)	0.020 (3)	0.017 (3)	−0.0002 (18)	0.008 (2)	0.000 (2)
C2	0.018 (2)	0.019 (3)	0.019 (2)	−0.0007 (19)	0.013 (2)	0.001 (2)
C3	0.014 (2)	0.018 (3)	0.019 (3)	−0.0001 (18)	0.010 (2)	−0.001 (2)
C4	0.018 (3)	0.020 (3)	0.016 (2)	−0.0001 (19)	0.011 (2)	0.002 (2)
C5	0.026 (3)	0.015 (3)	0.017 (2)	0.000 (2)	0.015 (2)	0.005 (2)
C6	0.023 (2)	0.016 (3)	0.018 (2)	−0.0038 (19)	0.013 (2)	−0.003 (2)
C7	0.025 (3)	0.010 (2)	0.019 (2)	0.0028 (19)	0.015 (2)	0.003 (2)
C8	0.025 (3)	0.015 (3)	0.013 (2)	0.000 (2)	0.012 (2)	−0.002 (2)
C9	0.019 (3)	0.020 (3)	0.013 (2)	−0.0007 (19)	0.010 (2)	−0.001 (2)
C10	0.015 (2)	0.015 (3)	0.020 (2)	0.0020 (18)	0.013 (2)	0.0033 (19)
C11	0.020 (2)	0.014 (2)	0.016 (2)	0.0004 (18)	0.013 (2)	−0.0021 (19)
C12	0.010 (2)	0.016 (3)	0.018 (2)	0.0019 (18)	0.0102 (19)	−0.002 (2)
La1	0.01520 (14)	0.01340 (16)	0.01582 (14)	0.00137 (10)	0.01041 (12)	0.00210 (11)
La2	0.01507 (14)	0.01329 (15)	0.01485 (14)	−0.00060 (10)	0.01008 (12)	−0.00145 (11)
O1	0.035 (2)	0.024 (2)	0.0153 (18)	−0.0022 (15)	0.0153 (16)	−0.0006 (15)
O2	0.0321 (19)	0.0115 (18)	0.0204 (18)	0.0018 (14)	0.0170 (16)	0.0048 (14)
O3	0.0296 (19)	0.0142 (18)	0.0211 (18)	0.0049 (14)	0.0148 (16)	0.0042 (14)
O4	0.0148 (16)	0.0217 (19)	0.0211 (18)	−0.0020 (13)	0.0123 (14)	−0.0078 (14)
O5	0.0188 (18)	0.027 (2)	0.0230 (18)	−0.0058 (14)	0.0140 (15)	−0.0108 (15)
O6	0.0202 (18)	0.031 (2)	0.0234 (19)	−0.0026 (14)	0.0158 (15)	−0.0061 (15)
O7	0.0218 (18)	0.025 (2)	0.0244 (19)	−0.0017 (14)	0.0116 (16)	−0.0084 (16)
O8	0.0267 (18)	0.0119 (18)	0.034 (2)	−0.0001 (14)	0.0208 (16)	−0.0045 (15)
O9	0.0268 (19)	0.033 (2)	0.0237 (19)	0.0059 (15)	0.0129 (16)	−0.0087 (16)
O10	0.0199 (17)	0.0202 (19)	0.033 (2)	0.0000 (14)	0.0170 (16)	0.0073 (16)
O11	0.0196 (17)	0.0160 (18)	0.0259 (18)	−0.0002 (14)	0.0126 (15)	0.0017 (15)
O12	0.0273 (18)	0.0150 (19)	0.0174 (17)	−0.0051 (14)	0.0115 (15)	−0.0027 (14)
O13	0.0247 (19)	0.031 (2)	0.028 (2)	0.0126 (15)	0.0197 (16)	0.0147 (16)
O14	0.0207 (18)	0.033 (2)	0.0245 (19)	0.0064 (15)	0.0181 (16)	0.0072 (16)
O15	0.0174 (17)	0.0201 (19)	0.0271 (19)	0.0030 (13)	0.0164 (15)	0.0106 (15)
O16	0.0309 (19)	0.0135 (18)	0.0235 (18)	−0.0071 (14)	0.0189 (16)	−0.0049 (14)
O17	0.037 (2)	0.0175 (19)	0.0171 (18)	0.0001 (15)	0.0166 (16)	0.0003 (15)
O18	0.0267 (18)	0.0165 (19)	0.0195 (17)	−0.0007 (14)	0.0164 (15)	−0.0011 (14)
O1W	0.0219 (19)	0.051 (2)	0.028 (2)	0.0068 (16)	0.0173 (16)	0.0164 (18)
O2W	0.035 (2)	0.022 (2)	0.0256 (19)	−0.0047 (16)	0.0143 (17)	−0.0026 (16)
O3W	0.027 (2)	0.052 (3)	0.039 (2)	−0.0224 (17)	0.0246 (18)	−0.0308 (19)
O5W	0.117 (5)	0.100 (5)	0.255 (10)	0.012 (4)	0.142 (6)	0.041 (6)
O4WA	0.103 (11)	0.045 (7)	0.053 (7)	−0.036 (7)	0.064 (8)	−0.019 (5)
O4WB	0.090 (9)	0.075 (8)	0.056 (6)	−0.033 (7)	0.047 (6)	0.005 (5)

### Geometric parameters (Å, °)

**Table:**

C1—O2	1.245 (5)	La1—O2W	2.563 (3)
C1—O1	1.266 (5)	La1—O9	2.679 (3)
C1—C2	1.525 (6)	La1—O3	2.698 (3)
C2—O3	1.424 (5)	La2—O5	2.478 (3)
C2—C3	1.524 (6)	La2—O14^ii^	2.488 (3)
C2—H2	0.9800	La2—O17^iii^	2.496 (3)
C3—O4	1.427 (5)	La2—O3W	2.505 (3)
C3—C4	1.531 (6)	La2—O11^iii^	2.529 (3)
C3—H3	0.9800	La2—O4	2.569 (3)
C4—O6	1.241 (5)	La2—O12^iv^	2.606 (3)
C4—O5	1.269 (5)	La2—O16^iii^	2.641 (3)
C5—O8	1.249 (5)	La2—O10^iii^	2.730 (3)
C5—O7	1.265 (5)	O3—H3A	0.8187
C5—C6	1.527 (6)	O4—H4	0.8211
C6—O9	1.430 (5)	O6—La1^v^	2.534 (3)
C6—C7	1.547 (6)	O9—H9	0.8167
C6—H6	0.9800	O10—La2^vi^	2.730 (3)
C7—O10	1.420 (5)	O10—H10A	0.8180
C7—C8	1.543 (6)	O11—La2^vi^	2.529 (3)
C7—H7	0.9800	O12—La2^vii^	2.606 (3)
C8—O11	1.258 (5)	O14—La2^viii^	2.488 (3)
C8—O12	1.260 (5)	O15—H15	0.8188
C9—O14	1.249 (5)	O16—La2^vi^	2.641 (3)
C9—O13	1.268 (5)	O16—H16	0.8184
C9—C10	1.516 (6)	O17—La2^vi^	2.496 (3)
C10—O15	1.426 (5)	O1W—H1W	0.8167
C10—C11	1.520 (6)	O1W—H2W	0.8174
C10—H10	0.9800	O2W—H4W	0.8180
C11—O16	1.429 (5)	O2W—H3W	0.8220
C11—C12	1.523 (6)	O3W—H6W	0.8155
C11—H11	0.9800	O3W—H5W	0.8177
C12—O17	1.241 (5)	O5W—H10W	0.8468
C12—O18	1.265 (5)	O5W—H9W	0.8478
La1—O7	2.458 (3)	O4WA—H4WA	0.8498
La1—O1	2.476 (3)	O4WA—H4WB	0.8499
La1—O1W	2.505 (3)	O4WA—H4WD	0.7409
La1—O13	2.519 (3)	O4WB—H4WC	0.8505
La1—O6^i^	2.534 (3)	O4WB—H4WD	0.8503
La1—O15	2.537 (3)		
			
O2—C1—O1	122.7 (4)	O1—La1—O3	59.64 (10)
O2—C1—C2	119.5 (4)	O1W—La1—O3	72.73 (10)
O1—C1—C2	117.8 (4)	O13—La1—O3	69.61 (10)
O3—C2—C3	113.3 (4)	O6^i^—La1—O3	129.38 (10)
O3—C2—C1	107.7 (4)	O15—La1—O3	109.80 (10)
C3—C2—C1	114.6 (4)	O2W—La1—O3	66.30 (10)
O3—C2—H2	106.9	O9—La1—O3	131.08 (10)
C3—C2—H2	106.9	O5—La2—O14^ii^	131.51 (10)
C1—C2—H2	106.9	O5—La2—O17^iii^	75.65 (10)
O4—C3—C2	113.9 (4)	O14^ii^—La2—O17^iii^	130.47 (10)
O4—C3—C4	107.5 (4)	O5—La2—O3W	144.32 (11)
C2—C3—C4	113.1 (4)	O14^ii^—La2—O3W	70.40 (10)
O4—C3—H3	107.3	O17^iii^—La2—O3W	69.74 (11)
C2—C3—H3	107.3	O5—La2—O11^iii^	80.05 (10)
C4—C3—H3	107.3	O14^ii^—La2—O11^iii^	101.25 (11)
O6—C4—O5	124.2 (4)	O17^iii^—La2—O11^iii^	126.38 (10)
O6—C4—C3	118.9 (4)	O3W—La2—O11^iii^	128.09 (11)
O5—C4—C3	116.8 (4)	O5—La2—O4	61.24 (9)
O8—C5—O7	124.6 (4)	O14^ii^—La2—O4	72.79 (10)
O8—C5—C6	117.5 (4)	O17^iii^—La2—O4	129.34 (10)
O7—C5—C6	117.8 (4)	O3W—La2—O4	140.56 (10)
O9—C6—C5	108.5 (4)	O11^iii^—La2—O4	72.77 (10)
O9—C6—C7	111.8 (4)	O5—La2—O12^iv^	76.47 (10)
C5—C6—C7	115.0 (4)	O14^ii^—La2—O12^iv^	78.72 (10)
O9—C6—H6	107.0	O17^iii^—La2—O12^iv^	68.33 (10)
C5—C6—H6	107.0	O3W—La2—O12^iv^	82.92 (11)
C7—C6—H6	107.0	O11^iii^—La2—O12^iv^	147.59 (10)
O10—C7—C8	108.0 (3)	O4—La2—O12^iv^	76.44 (10)
O10—C7—C6	111.6 (4)	O5—La2—O16^iii^	76.20 (10)
C8—C7—C6	109.6 (3)	O14^ii^—La2—O16^iii^	149.60 (10)
O10—C7—H7	109.2	O17^iii^—La2—O16^iii^	60.38 (10)
C8—C7—H7	109.2	O3W—La2—O16^iii^	93.63 (10)
C6—C7—H7	109.2	O11^iii^—La2—O16^iii^	67.75 (10)
O11—C8—O12	125.4 (4)	O4—La2—O16^iii^	125.67 (9)
O11—C8—C7	117.3 (4)	O12^iv^—La2—O16^iii^	126.16 (9)
O12—C8—C7	117.2 (4)	O5—La2—O10^iii^	136.93 (10)
O14—C9—O13	124.9 (4)	O14^ii^—La2—O10^iii^	72.81 (10)
O14—C9—C10	117.5 (4)	O17^iii^—La2—O10^iii^	118.40 (10)
O13—C9—C10	117.6 (4)	O3W—La2—O10^iii^	70.51 (11)
O15—C10—C9	109.0 (3)	O11^iii^—La2—O10^iii^	58.50 (9)
O15—C10—C11	111.5 (4)	O4—La2—O10^iii^	111.26 (10)
C9—C10—C11	110.6 (4)	O12^iv^—La2—O10^iii^	146.07 (9)
O15—C10—H10	108.6	O16^iii^—La2—O10^iii^	77.54 (10)
C9—C10—H10	108.6	C1—O1—La1	131.0 (3)
C11—C10—H10	108.6	C2—O3—La1	121.9 (3)
O16—C11—C10	110.8 (4)	C2—O3—H3A	103.0
O16—C11—C12	108.7 (3)	La1—O3—H3A	114.7
C10—C11—C12	112.6 (4)	C3—O4—La2	119.8 (2)
O16—C11—H11	108.2	C3—O4—H4	108.6
C10—C11—H11	108.2	La2—O4—H4	121.1
C12—C11—H11	108.2	C4—O5—La2	127.0 (3)
O17—C12—O18	125.4 (4)	C4—O6—La1^v^	135.2 (3)
O17—C12—C11	118.4 (4)	C5—O7—La1	131.3 (3)
O18—C12—C11	116.2 (4)	C6—O9—La1	121.6 (2)
O7—La1—O1	78.94 (11)	C6—O9—H9	104.2
O7—La1—O1W	76.88 (11)	La1—O9—H9	104.9
O1—La1—O1W	75.70 (11)	C7—O10—La2^vi^	123.3 (2)
O7—La1—O13	123.42 (11)	C7—O10—H10A	108.1
O1—La1—O13	76.42 (10)	La2^vi^—O10—H10A	126.4
O1W—La1—O13	140.85 (11)	C8—O11—La2^vi^	131.4 (3)
O7—La1—O6^i^	74.88 (11)	C8—O12—La2^vii^	133.4 (3)
O1—La1—O6^i^	145.67 (10)	C9—O13—La1	126.5 (3)
O1W—La1—O6^i^	77.03 (10)	C9—O14—La2^viii^	143.6 (3)
O13—La1—O6^i^	137.11 (10)	C10—O15—La1	123.8 (2)
O7—La1—O15	115.51 (10)	C10—O15—H15	110.8
O1—La1—O15	136.82 (10)	La1—O15—H15	120.3
O1W—La1—O15	145.22 (10)	C11—O16—La2^vi^	122.1 (2)
O13—La1—O15	61.51 (10)	C11—O16—H16	105.4
O6^i^—La1—O15	75.61 (10)	La2^vi^—O16—H16	126.4
O7—La1—O2W	142.04 (11)	C12—O17—La2^vi^	128.9 (3)
O1—La1—O2W	125.34 (11)	La1—O1W—H1W	111.2
O1W—La1—O2W	81.70 (12)	La1—O1W—H2W	127.1
O13—La1—O2W	92.68 (11)	H1W—O1W—H2W	105.8
O6^i^—La1—O2W	69.95 (11)	La1—O2W—H4W	116.7
O15—La1—O2W	69.01 (10)	La1—O2W—H3W	117.0
O7—La1—O9	60.31 (10)	H4W—O2W—H3W	104.7
O1—La1—O9	87.05 (11)	La2—O3W—H6W	128.7
O1W—La1—O9	136.24 (11)	La2—O3W—H5W	124.9
O13—La1—O9	68.26 (11)	H6W—O3W—H5W	105.5
O6^i^—La1—O9	98.66 (11)	H10W—O5W—H9W	105.7
O15—La1—O9	69.45 (10)	H4WA—O4WA—H4WB	105.2
O2W—La1—O9	138.45 (11)	H4WA—O4WA—H4WD	106.4
O7—La1—O3	133.15 (10)	H4WC—O4WB—H4WD	105.1

Symmetry codes: (i) *x*, −*y*+3/2, *z*+1/2; (ii) *x*−1, −*y*+3/2, *z*−1/2; (iii) *x*−1, *y*, *z*−1; (iv) −*x*, *y*−1/2, −*z*+3/2; (v) *x*, −*y*+3/2, *z*−1/2; (vi) *x*+1, *y*, *z*+1; (vii) −*x*, *y*+1/2, −*z*+3/2; (viii) *x*+1, −*y*+3/2, *z*+1/2.

### Hydrogen-bond geometry (Å, °)

**Table:**

*D*—H···*A*	*D*—H	H···*A*	*D*···*A*	*D*—H···*A*
O3—H3A···O8^iv^	0.82	1.87	2.677 (4)	169
O4—H4···O18^vii^	0.82	1.80	2.619 (4)	174
O9—H9···O16	0.82	2.46	3.177 (5)	147
O10—H10A···O8^ix^	0.82	1.94	2.745 (4)	167
O15—H15···O2^iv^	0.82	1.82	2.632 (4)	175
O16—H16···O4WA	0.82	1.89	2.667 (9)	158
O16—H16···O4WB	0.82	1.94	2.708 (9)	157

Symmetry codes: (iv) −*x*, *y*−1/2, −*z*+3/2; (vii) −*x*, *y*+1/2, −*z*+3/2; (ix) −*x*, −*y*+2, −*z*+2.
